# Recurrent Alternate Parthenogenesis in the Common Smooth-Hound Shark (*Mustelus mustelus*) with Additional Cases and Further Evidence for a Putative Adaptive Reproductive Strategy

**DOI:** 10.3390/ani16101423

**Published:** 2026-05-07

**Authors:** Simona Sciuto, Giuseppe Esposito, Flavio Gagliardi, Matteo Riccardo Di Nicola, Paolo Pastorino, Nadia Ruiu, Giulia Milanese, Nicole Kube, Oscar Di Santo, Marino Prearo, Pier Luigi Acutis, Silvia Colussi

**Affiliations:** 1 Istituto Zooprofilattico Sperimentale del Piemonte, Liguria e Valle d’Aosta, 10154 Turin, Italy; simona.sciuto@izsplv.it (S.S.); matteoriccardo.dinicola@izsplv.it (M.R.D.N.); paolo.pastorino@izsplv.it (P.P.); giulia.milanese@izsplv.it (G.M.); marino.prearo@izsplv.it (M.P.); pierluigi.acutis@izsplv.it (P.L.A.); silvia.colussi@izsplv.it (S.C.); 2 Acquario di Cala Gonone, Cala Gonone, 08022 Dorgali, Italy; 3 Panaque s.r.l., 00144 Rome, Italy; 4 Ocean Museum Germany Foundation, Katharinenberg 14-20, 18439 Stralsund, Germany

**Keywords:** captive breeding, elasmobranch conservation, facultative parthenogenesis, molecular confirmation, reproductive ecology

## Abstract

A new case of parthenogenesis in the common smooth-hound shark (*Mustelus mustelus*), recorded at the Cala Gonone Aquarium (central-eastern Sardinia, Italy) in April 2024, is described as an extension of previous observations. The event involves the production of two offspring originating from a single parthenogenetic reproductive episode, further expanding the available evidence. Genetic analyses excluded paternal contribution, supporting the occurrence of facultative parthenogenesis. The data confirm the recurrence of parthenogenetic reproduction in *M. mustelus* under captive conditions and document an alternation between two adult females across successive parthenogenetic reproductive events. This recurring pattern supports the hypothesis of a structured reproductive dynamic and suggests the involvement of individual-specific or environmentally mediated factors. These findings contribute to the current understanding of facultative parthenogenesis in elasmobranchs by providing additional empirical evidence across successive reproductive events. The results also offer further insight into the potential adaptive significance of this reproductive mode under conditions of limited mate availability, with implications for the management and conservation of captive populations.

## Simple Summary

Parthenogenesis is a type of reproduction in which females can produce offspring without male fertilisation. We report a new case in the common smooth-hound shark at the Cala Gonone Aquarium in Italy, involving multiple offspring from a single reproductive event. Observations show that two adult females produced parthenogenetic offspring in succession, revealing a recurring reproductive pattern. These findings provide further evidence of facultative parthenogenesis in sharks and suggest that individual traits or environmental factors may influence its occurrence. Understanding this reproductive strategy is important for managing captive sharks and supporting conservation efforts, particularly when mate availability is limited.

## 1. Introduction

Successful reproduction is crucial for species persistence and is particularly important for threatened taxa. The ability of elasmobranchs to reproduce by parthenogenesis is now widely recognised, and facultative parthenogenesis has previously been documented in the common smooth-hound (*Mustelus mustelus* (Linnaeus, 1758), Carcharhiniformes: Triakidae), representing the first confirmed case of this phenomenon within the genus [1]. The genus *Mustelus* comprises 29 species of benthic sharks inhabiting temperate and tropical continental-shelf waters worldwide [2]. In the Mediterranean region, *M. mustelus* coexists with the blackspotted smooth-hound (*Mustelus punctulatus*, Risso, 1826) and the starry smooth-hound (*Mustelus asterias*, Cloquet, 1819), which display distinct reproductive traits and cycles. The common smooth-hound shark is currently listed as Endangered (EN A2bd) on the global IUCN Red List of Threatened Species [2]. In the Italian IUCN Red List of Vertebrates, demographic models predicted a 50% population decline over a 20-year period under current fishing pressure, and the species is assessed as Endangered (EN A3d) [3]. This species is frequently caught as bycatch in both bottom and pelagic trawl fisheries and, seasonally, is targeted by artisanal coastal fisheries, particularly in the northern Adriatic Sea [3,4,5]. For example, at the Chioggia fish market, one of Italy’s largest landing sites, an estimated 70% of males and 90% of females landed are sexually immature [3].

Parthenogenesis, defined as the development of an embryo from an unfertilised oocyte, is widespread in invertebrates but remains rare in vertebrates, in which obligate parthenogenesis is largely restricted to squamate reptiles, and facultative parthenogenesis has been documented in birds, non-avian reptiles, and elasmobranchs [6,7]. Natural facultative parthenogenesis has not been demonstrated in mammals, where genomic imprinting creates a fundamental developmental barrier to the production of viable offspring from an exclusively maternal genome [8,9].

Over the past two decades, the increasing use of microsatellite and genomic analyses has shown that some births formerly attributed to undocumented mating or retained sperm are instead true cases of facultative parthenogenesis, substantially expanding recognition of this phenomenon across vertebrate lineages [7,10]. In elasmobranchs, this distinction is particularly important because long-term sperm storage is a recognised component of female reproductive biology, occurring in the oviducal gland across chondrichthyans and having been documented specifically in the common smooth-hound [11,12].

Consequently, putative cases of parthenogenesis in sharks require genetic evidence excluding paternal contribution and demonstrating the marked homozygosity expected under automictic development [1,10,13].

The first genetically verified case of parthenogenesis in a cartilaginous fish was reported in the bonnethead (*Sphyrna tiburo* (Linnaeus, 1758)), in which DNA analysis excluded paternal contribution and supported the automictic origin of the offspring [10].

Subsequent reports showed that this phenomenon is not restricted to a single elasmobranch lineage, as annual recurrent parthenogenesis was later documented in a captive zebra shark (*Stegostoma tigrinum* (Forster, 1781)) over four consecutive years [14]. In the same species, an intra-individual switch from sexual to parthenogenetic reproduction was subsequently demonstrated, and a sexually produced daughter also began reproducing parthenogenetically at the onset of maturity despite no prior mating history [6]. In whitespotted bamboosharks (*Chiloscyllium plagiosum* (Anonymous [Bennett], 1830)), facultative parthenogenesis produced multiple viable offspring that survived for more than five years, demonstrating that shark parthenogens are not necessarily short-lived or developmentally inviable [15]. Later, second-generation facultative parthenogenesis was documented in the same species, providing the first genetically confirmed evidence of this phenomenon recurring across two generations in a vertebrate lineage [16]. Additional shark reports further broadened the known spectrum of outcomes, including multiple pups produced parthenogenetically by a captive swellshark (*Cephaloscyllium ventriosum* (Garman, 1880)) and a case of parthenogenesis in a whitetip reef shark (*Triaenodon obesus* (Rüppell, 1837)), associated with a reduction in ploidy [13,17]. Moreover, in whitespotted bamboosharks, sexually fertilised and parthenogenetic offspring were shown to occur within the same clutch following artificial insemination, indicating that these two reproductive pathways can coexist within a single reproductive cycle [18]. Finally, a zebra shark case documented parthenogenesis in the presence of reproductively mature conspecific males, indicating that the phenomenon is not necessarily restricted to conditions of strict male absence [19].

Taken together, these studies indicate that facultative parthenogenesis in sharks is more variable than initially assumed and cannot be interpreted simply as an incidental consequence of prolonged isolation from males [6,18,19]. In most vertebrate systems, facultative parthenogenesis is interpreted as a form of automixis, and in sharks, the high homozygosity observed in parthenogenetic offspring is generally consistent with that expectation, especially under terminal fusion models [1,6,10,13]. Because the automictic development usually causes a pronounced reduction in heterozygosity, it may expose recessive deleterious variants and thereby contribute to developmental abnormalities, embryonic mortality, or reduced postnatal viability [1,13,20]. Evidence from the critically endangered smalltooth sawfish (*Pristis pectinata* (Latham, 1794)) further demonstrated that facultative parthenogenesis can also occur in wild elasmobranch populations, not only in captivity, and may therefore be relevant in natural contexts where mate encounter rates are reduced [21]. Nevertheless, although facultative parthenogenesis may provide short-term reproductive assurance under specific circumstances, it is unlikely to compensate for the long-term genetic benefits of sexual reproduction because it does not restore paternal allelic input and is typically associated with elevated homozygosity [7,20,21].

Within this framework, the common smooth-hound shark is of particular interest, as the first confirmed case of recurrent facultative parthenogenesis in this species was recently reported in a captive population housed at the Cala Gonone Aquarium [1]. That study showed that parthenogenetic offspring were consistently homozygous at all analysed loci, a pattern compatible with terminal fusion automixis, and also excluded long-term sperm storage as the explanation for the observed births [1]. However, the recurrence of parthenogenesis within individual females, the possible production of multiple offspring in a single reproductive event, and the viability of parthenogenetic juveniles remain incompletely characterised in this species and in sharks more broadly [1,6,15]. The present study therefore provides additional information on the expression of facultative parthenogenesis in *M. mustelus* by documenting (i) multiple offspring arising from a single parthenogenetic reproductive event, (ii) a second independent occurrence of alternation between two adult females across successive parthenogenetic events, and (iii) an extended observation period compared to previous reports. In this way, it refines and expands the comparative framework necessary to interpret the biological and conservation significance of this rare reproductive phenomenon in elasmobranchs [1,7].

## 2. Materials and Methods

### 2.1. Husbandry Conditions and Study Animals

All husbandry procedures, including tank systems, water quality parameters, photoperiod, and feeding protocols, followed the methods described in Esposito et al. [1], as this study represents an update on the same captive population. Adult specimens were maintained in the largest exhibition tank of the Cala Gonone Aquarium (350,000 L; 350 m^3^), equipped with autonomous Life Support Systems (LSS) for the control of physical and chemical parameters.

Water temperature ranged seasonally from 17.3 ±  0.3 °C (winter) to 27.5 ±  0.4 °C (summer), while salinity and pH remained relatively constant at 37.0 ±  0.4 and 8.2 ±  0.1, respectively (mean ±  SD). Photoperiod averaged 10.0 ±  2.8 h of artificial light, with additional variability due to natural light entering through a skylight. The tank is structured into three interconnected sectors at different depths (3.20 m, 3.65 m, 4.15 m), combining natural and artificial illumination (LED system, 20,000 K white and 450 nm royal blue). Continuous water renewal is ensured through seawater intake and a dedicated supply system with mechanical filtration and storage capacity, allowing long-term stable maintenance of environmental conditions. Feeding protocols followed the established routine described in Esposito et al. [1].

The two adult female *Mustelus mustelus* were collected alive as juveniles in 2010 from the Gulf of Orosei (40°14′45.40″ N, 9°40′07.64″ E; central-eastern Sardinia, Italy) and were maintained under controlled conditions at the Cala Gonone Aquarium thereafter.

From their introduction onwards, the females have never been in contact with males of the same species or genus, and no additional sharks have been added to the tank, except for a single female nursehound (*Scyliorhinus stellaris* (Linnaeus, 1758)).

At the time of the first recorded parthenogenetic event, both females were 18 years old [1]. On 18 April 2024, two new pups were stillborn; their biometrics, including total length (TL), were measured using a measuring tape (cm), and total weights (g) were recorded with a VEVOR Digital Balance (0.01 g precision).

### 2.2. Molecular Analyses

Genomic DNA was isolated from dorsal muscle tissues using the ReliaPrep gDNA Tissue Miniprep System kit (Promega, Madison, WI, USA). Extractions were performed in triplicate for each sample. DNA purity and concentration were evaluated using UV absorbance measured with a NanoDrop spectrophotometer (Thermo Fisher Scientific, Waltham, MA, USA), based on the A260/280 and A260/230 ratios.

A panel of 13 species-specific microsatellite markers was employed for individual genotyping, following the protocol described by Marino et al. [22]. The main characteristics of the microsatellite loci are reported in Supplementary Table S1.

PCR amplification was conducted in simplex reactions with a final volume of 10 µL, using the following mixture: 5 µL of Master Mix, 0.187 µL of forward primer (10 µM), 0.187 µL of reverse primer (10 µM), 3.6 µL of nuclease-free water, and 1 µL of DNA template. The thermal cycling conditions included an initial denaturation step at 95 °C for 5 min, followed by 30 cycles of denaturation at 94 °C for 30 s, annealing at 57 °C for 1 min 30 s, and extension at 72 °C for 1 min, with a final extension at 72 °C for 30 min.

Fragment analysis was carried out using LIZ 500 (Applied Biosystems) as the internal size standard, with 1 µL of DNA diluted 1:50. Samples were processed on an automated 3130XL Genetic Analyzer (Applied Biosystems, Foster City, CA, USA), and allele sizes were scored using GeneMapper software v6.0 (Thermo Fisher Scientific, Waltham, MA, USA).

### 2.3. Ethical Statement

No animals were sacrificed for this study. The genetic analyses were performed *postmortem* on tissue samples collected from pups that were found dead at birth, and the results were compared with those reported by Esposito and colleagues [1]. All procedures complied with institutional and national guidelines for the ethical use of animals in research.

The Cala Gonone Aquarium operated under Zoo Legislation [23]. This decree was published in the Official Gazette No. 100 on 2 May 2005.

## 3. Results

The biometric data recorded at the first parthenogenetic event and at the time of the present update, including Juveniles 1 to 4 already described by Esposito et al. [1], are summarised in Table 1.

Adult specimens were reared in tanks equipped with autonomous water treatment systems (Life Support System, LSS) that allowed control of the main physicochemical parameters (see Section 2.1). The main water parameters have remained stable since the previous monitoring period, with temperature (°C), salinity, and pH values comparable to those reported by Esposito et al. [1]. Therefore, environmental conditions were considered consistent during the subsequent birth events.

Juvenile no. 1 was born from one of the two adult females in 2016 but was not retained for further investigation (Table 1). Three additional birth events occurred in 2020, 2021, and 2023. Among these, only one juvenile (no. 3, born in 2021) survived long-term, reaching three years of age before dying in September 2024. However, in the months preceding death, this individual exhibited inappetence and a rapid decline in body mass, decreasing from 1820 g to 1248 g, representing an approximate loss of 31% (Table 1). *Postmortem* investigations to determine the cause of death are still ongoing at the time of writing.

All juvenile sharks were females and displayed bite marks on various body regions [1], likely resulting from intraspecific interactions and possibly contributing to mortality in juveniles Nos. 1, 2, and 4. The latter, born in 2023, also exhibited body deformities as well as deep cranial wounds [1].

Both adult females were maintained in the absence of conspecific males and continued to grow after the measurements reported by Esposito et al. [1]. The first female died in October 2025, followed by the second female in February 2026. Adult No. 1 increased from 137.2 cm TL and 17,560 g to 157.0 cm TL and 18,214 g, while Adult No. 2 grew from 142.3 cm TL and 19,230 g to 151.5 cm TL and 20,142 g (Table 1).

**Figure 1:**
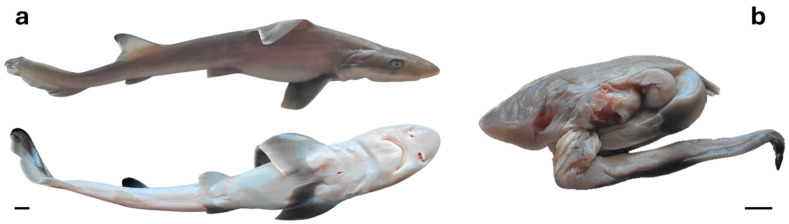
Female specimens of *Mustelus mustelus* born through parthenogenesis at the Cala Gonone Aquarium in April 2024, both stillborn: (**a**) fully developed juvenile; (**b**) premature, underdeveloped individual. Scales bar: 1 cm. Photo credits: G. Esposito.

**Figure 2:**
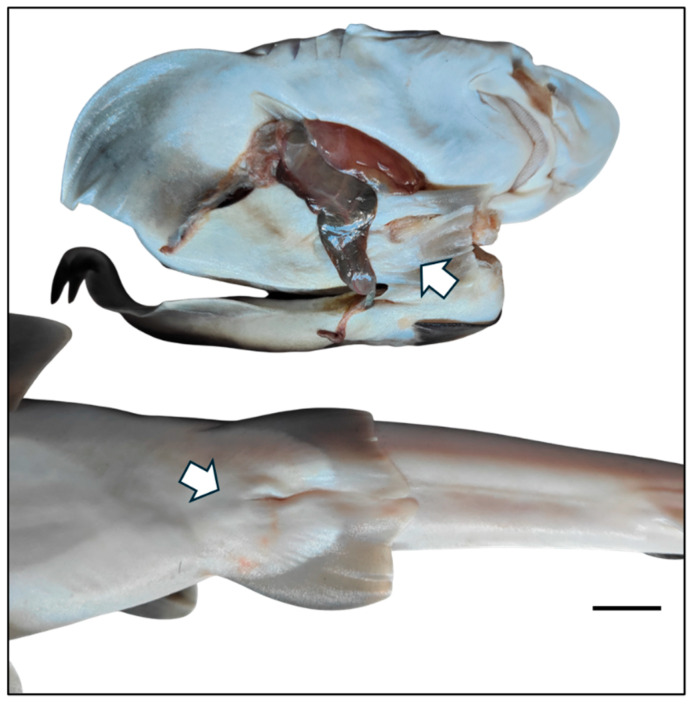
Macroscopic detail highlighting the female sex of two parthenogenetic *Mustelus mustelus*. White arrows indicate the external genital structures confirming the female sex. Scale bar: 1 cm. Photo credits: G. Esposito.

Two additional birth events occurred in 2024, resulting in the birth of juvenile Nos. 5 and 6 (Figure 1 and Figure 2; Table 1). Both sharks were found dead within 24 h. Juvenile No. 5 measured 41.2 cm in TL and weighed 237.4 g, whereas juvenile No. 6 weighed 133.1 g; in the latter case, TL could not be measured as the specimen was a premature shark and could not be fully extended (Figure 1 and Figure 2). However, an estimate of TL was obtained through image analysis using ImageJ software (ImageJ, version 1.55s, National Institutes of Health, USA).

Loci McaB5, Mh25, McaB35, MaD2X, and McaB26 were found in homozygosity in juveniles 5 and 6, thus confirming parthenogenesis (Table 2). Mh25, McaB35, and McaB26 were also informative for maternity. Amplification of McaB26 in juvenile specimen No. 5 failed after repeated attempts. Both juvenile specimens Nos. 5 and 6 were attributed to adult female No. 2 (Table 2).

## 4. Discussion

The observed biometric increments are modest. In the wild, this species can reach substantially larger sizes, with reported maximum lengths up to 200 cm TL and a maximum age of 24 years [24,25,26]. Compared to the measurements reported by Esposito et al. [1], the two adult *M. mustelus* females exhibited a modest but heterogeneous increase in total length (+6–14%) and a limited increase in body weight (+4–5%). These increments indicate that somatic growth, although substantially attenuated, may persist in long-term captive elasmobranchs. Comparable long-term growth records have been documented in public aquaria for several species (e.g., nurse sharks (*Ginglymostoma cirratum*, Bonnaterre, 1788)) [27,28,29], but species-specific long-term studies for *M. mustelus* are lacking and warrant further investigation. This pattern is consistent with the species’ life-history traits, characterised by slow growth, late maturation, and prolonged longevity, and reflects the reduced growth rates typically observed in elasmobranchs maintained under stable controlled conditions [30].

In contrast to the slow but steady growth observed in the adult females, the captive-born juveniles exhibited high early mortality, with five out of six individuals dying within a few days of birth. Only Juvenile 3, born in 2021, survived for three years, although it ultimately died after experiencing inappetence and rapid weight loss.

However, the early mortality observed in the offspring cannot be unequivocally attributed to parthenogenesis. Observations from *Mustelus asterias* maintained in captivity indicate that low pup numbers, stillbirths, and low neonatal survival are relatively frequent during the initial reproductive years, even in sexually reproducing females (Kube N., pers. comm.). Such patterns, reported across multiple institutions, suggest that early reproductive failure may be strongly influenced by husbandry-related factors rather than by reproductive mode alone (Kube N., pers. comm.).

Initial total lengths (TL) and body weights (W) of pups varied between birth events, with later births in 2024 producing slightly larger neonates (Juvenile No. 5: 41.2 cm, 237.4 g; Juvenile No. 6: 26.9 cm, 133.1 g) compared to earlier ones (e.g., Juvenile No. 2: 33.7 cm, 131.5 g). These values are consistent with the range reported for pups of *M. mustelus* in wild populations, with size at birth estimated at approximately 34–43.5 cm TL and body mass values of 80–234 g based on full-term embryos and early pups [31,32]. Overall, the observed biometrics are consistent with size ranges for the species [33], which document considerable intraspecific variability in total length across populations.

These observations suggest that, while adult *M. mustelus* are capable of continued somatic growth under stable captive conditions, the survival of parthenogenetic pups remains low, probably due to their low genetic variability, even when environmental parameters are maintained within optimal ranges. However, the available evidence does not allow clear discrimination between potential genetic constraints associated with parthenogenesis and environmental or management-related causes.

In *M. asterias*, low neonatal survival has been documented regardless of reproductive mode, while improvements in captive conditions (particularly higher water temperatures and increased tank volume) have been linked to markedly enhanced offspring survival and sustained breeding success (Kube N., pers. comm.). These findings are consistent with recent research indicating that North Atlantic sharks utilise coastal nursery areas characterised by warmer and more stable environmental conditions [34], which are thought to promote early-life survival. Such ecological requirements may explain why suboptimal captive conditions can disproportionately affect neonatal survival and may also contribute to the occurrence of atypical reproductive outcomes.

However, caution is required when extrapolating these findings to *M. mustelus*, for which species-specific data remain scarce. In parallel, a growing body of evidence indicates that facultative parthenogenesis in elasmobranchs may entail intrinsic fitness costs [6,10,35]. For example, parthenogenetic zebra sharks (*Stegostoma tigrinum*) display reduced growth rates, behavioural abnormalities, and decreased longevity relative to sexually produced individuals [35]. Similarly, an early study on the bonnethead (*Sphyrna tiburo*) highlighted the genetic consequences of automictic parthenogenesis, notably reduced heterozygosity [10].

In this context, the presence of bite marks observed in several juveniles (Nos. 1, 2, and 4) suggests that intraspecific interactions may have further increased vulnerability during early developmental stages. Such social dynamics, while not uncommon in captive aquatic systems [1] and consistent with the documented capacity for social interaction in elasmobranchs [36], could represent an additional stressor acting on individuals exhibiting reduced physiological robustness.

Although the survival of Juvenile No. 3 demonstrates that medium-term growth is possible, the overall pattern indicates that early life stages represent a critical bottleneck.

Overall, these findings support a multifactorial interpretation of mortality, likely resulting from the interaction between intrinsic constraints associated with parthenogenesis and extrinsic factors, including social interactions within the captive environment. This interpretation is consistent with emerging evidence indicating that parthenogenetic elasmobranchs may exhibit reduced fitness, potentially amplifying sensitivity to environmental and social stressors [35].

The microsatellite profiles of the newly born individuals, compared with those of the putative mothers, confirmed their parthenogenetic origin. Alternate recurrence between the two adult females in producing parthenogenetic offspring is also confirmed, since juvenile Nos. 2 and 4 were attributed to the adult female No. 1, while juvenile Nos. 3, 5, and 6 were attributed to the adult female No. 2. In particular, juveniles Nos. 5 and 6 were produced within the same parturition event (April 2024). Moreover, in *M. mustelus*, we report for the first time the production of multiple offspring through parthenogenesis from the same mother, a pattern previously documented in other elasmobranchs, such as the whitespotted bambooshark [15], thereby placing our findings within a broader comparative context.

This alternating pattern raises the question of whether coordinated physiological or behavioural mechanisms may regulate the timing of these reproductive events. The observed alternation in parthenogenetic births between the two females raises the question of whether intrinsic physiological or environmental mechanisms may influence the timing of facultative parthenogenesis. At present, there is no evidence for any coordinated hormonal or social signalling mechanism regulating reproductive alternation among individuals in elasmobranchs. Rather, available evidence indicates that reproduction in chondrichthyans is primarily governed by individual endocrine cycles. Steroid hormones such as oestradiol (E2), testosterone (T), and progesterone (P4) play key roles in regulating vitellogenesis, follicular development, ovulation, and parturition, and their temporal dynamics provide reliable indicators of reproductive status [37,38]. For instance, in captive zebra sharks, E2 increases prior to follicular development and declines with follicular regression, while T peaks during the egg-laying period, highlighting tight hormonal control of reproductive timing [38].

Importantly, these endocrine pathways are highly sensitive to environmental conditions. Temperature, in particular, has been shown to modulate steroid hormone profiles and reproductive timing. In narrownose smooth-hound (*Mustelus schmitti*, Springer, 1939), T and E2 exhibit opposite temperature-dependent patterns, with T increasing markedly at higher temperatures and peaking near parturition, followed by a rise in P4 associated with ovulation [39]. Such findings suggest that environmental cues can trigger or shift reproductive events through hormonally mediated mechanisms. More broadly, elasmobranch reproductive biology is known to be vulnerable to external stressors, including environmental changes and anthropogenic pressures, which can alter physiological processes underlying reproduction [40].

Taken together, this evidence supports the interpretation that the alternation observed in *M. mustelus* is more plausibly explained by asynchronous reproductive cycles and individual-specific endocrine states, potentially modulated by environmental conditions, rather than by any form of inter-individual coordination or signalling mechanism.

Maintaining shark populations is essential for marine ecosystem stability, particularly given the key role of elasmobranchs as apex or mesopredators shaping trophic dynamics. In this context, reproductive processes such as facultative parthenogenesis may have important implications for genetic structure and demographic trajectories in natural populations, especially under conditions of reduced mating opportunities. The present findings further highlight the value of controlled environments in elucidating rare reproductive mechanisms, as aquaria allow the integration of long-term biological monitoring and genetic analyses that are not feasible in wild populations. In particular, the detection and confirmation of parthenogenetic events, as well as their reproductive patterns (e.g., alternating births observed in this study), would be extremely difficult to document in situ without complete population sampling.

Therefore, while aquaria are not the primary focus of this study, they provide a crucial observational framework that complements and strengthens the interpretation of reproductive processes relevant to conservation biology. In particular, aquaria have become increasingly important for the study and conservation of threatened species, as they enable detailed investigations of reproductive biology and life-history traits that are often inaccessible in wild populations, thereby contributing valuable insights for the development of conservation strategies in situ [41]. In endangered species, parthenogenesis may lead to a decrease in genotypic diversity and population decline, which is likely to diminish the ability to survive in the environment [10]. At the same time, if shark populations decline, parthenogenesis could represent a reproductive adaptation for females in response to the prolonged absence of mates in the marine environment.

Parthenogenesis, although associated with reduced genetic variability compared to sexual reproduction, has been interpreted across multiple systems as a contingent reproductive strategy that enables persistence in marginal environments or under conditions of reproductive isolation. For example, parthenogenetic lizard species (e.g., *Aspidoscelis* spp.) are often associated with disturbed (“disclimax”) environments and occur in isolation from closely related sexual species [42], conditions that may facilitate their establishment and spread. In many cases, these lineages originate through hybridisation and are linked to shifts in geographical distribution, particularly in newly available or environmentally unstable habitats, where parthenogenesis may contribute to the rapid establishment and stabilisation of successful genotypes [43,44,45]. Facultative parthenogenesis has likewise been reported in vertebrates under conditions of mate limitation or reproductive isolation, highlighting its role as an alternative reproductive pathway rather than a rare evolutionary anomaly [46].

Moreover, genetically confirmed cases of parthenogenesis in elasmobranchs, including blacktip shark (*Carcharhinus limbatus*, Valenciennes, 1839) and *S. tigrinum*, demonstrate that this reproductive mode occurs in chondrichthyan fishes and is likely more widespread than previously assumed. These cases, along with additional reports in other shark species, indicate that automictic parthenogenesis can arise in the absence of males and has been associated with conditions of prolonged isolation or limited mating opportunities, suggesting a flexible reproductive response in these lineages [6,10,15,47].

In this context, parthenogenesis should not necessarily be regarded as an evolutionary “dead end” but rather as a “stop-gap” strategy that may ensure short-term population persistence despite potential long-term constraints.

These considerations are particularly relevant for in situ conservation scenarios, where severe reductions in male abundance may limit mating opportunities and disrupt effective reproductive connectivity. As also highlighted by Feldheim and colleagues [19,29], parthenogenesis in elasmobranchs can occur not only in the absence of males but also in the presence of reproductively active males, suggesting that it may be associated with unsuccessful mating, cryptic fertilization processes, or asynchronous reproductive timing rather than strict mate absence. In such contexts, facultative parthenogenesis may allow isolated females to produce offspring and contribute to short-term demographic persistence under extreme mate limitation. However, it does not represent a reliable mechanism for replacing sexual reproduction or sustaining long-term population recovery, as it does not restore gene flow or genetic diversity. Consequently, population persistence and recovery in depleted systems are expected to depend primarily on the re-establishment of functional mating systems.

The overall incidence of parthenogenesis in *M. mustelus* in the wild remains unknown. In Europe, *M. mustelus* is currently maintained in relatively low numbers across a limited number of institutions, which constrains the availability of long-term reproductive and genetic datasets and underscores the importance of documenting rare reproductive events when they occur (Kube N., pers. comm).

Feldheim and colleagues [19] documented a case of parthenogenesis in a female zebra shark despite the presence of conspecific males. Although parthenogenesis in *Mustelus* has been reported primarily in females housed in captivity [1], it would be valuable to investigate whether this event can also occur as an alternative reproductive strategy when males are present.

## 5. Conclusions

The confirmation of parthenogenesis in *Mustelus mustelus* represents a significant contribution to the growing evidence that this reproductive mode may occur across a broader range of elasmobranch taxa than previously assumed. Similar to observations in other shark species, parthenogenesis in *M. mustelus* appears to manifest under captive conditions, supporting the hypothesis that it may represent a facultative reproductive strategy, particularly in contexts of limited mate availability.

The demonstration of recurrent parthenogenesis in this study further suggests that this reproductive capability may not be an exceptional or isolated phenomenon in smooth-hound sharks. In particular, the observed alternation in the maternal role between the two adult females constitutes a novel and intriguing finding, indicating that parthenogenetic activation may be influenced by underlying biological or environmental factors rather than being restricted to a single individual.

Early mortality of offspring could not be unequivocally attributed to parthenogenesis itself. Evidence from other *Mustelus* species maintained in captivity indicates that low neonatal survival may also occur in sexually reproducing females, particularly during the initial reproductive years, and may be strongly influenced by husbandry conditions. Consequently, the fitness consequences of parthenogenesis in *M. mustelus* remain difficult to assess, and no definitive conclusions can yet be drawn regarding the relative viability of parthenogenetic versus sexually produced offspring.

Given the limited number of *M. mustelus* individuals currently maintained in European aquaria and the scarcity of long-term reproductive and genetic datasets, the documentation of rare reproductive events is of particular importance. Comprehensive genetic analyses, including parentage assessments, combined with standardised monitoring of environmental parameters, are essential to improve our understanding of this phenomenon.

These findings highlight the need for further multidisciplinary investigations, integrating genetics, reproductive physiology, and detailed husbandry data, to elucidate the mechanisms and potential triggers of parthenogenesis in smooth-hound sharks. Moreover, comparative studies across shark species, conducted both in captivity and in natural populations, will be crucial to determine whether parthenogenesis confers any adaptive value and to clarify its potential implications for population structure, genetic diversity, and long-term conservation of elasmobranchs, particularly through the application of integrative approaches such as population-wide parentage analyses, long-term individual monitoring, and emerging non-invasive tools, which may improve the detection of rare parthenogenetic events in wild populations.

## Figures and Tables

**Table 1:** Biometric data of two *Mustelus mustelus* females reared in captivity. Details of specimens introduced and born at the Cala Gonone Aquarium (Italy). Modified from Esposito et al. [1].

Shark	Introduction Year (I)/Birth Year (B)	Current Status	Female	Reproductive Mode	Age	TL (cm)	W (g)
Female no. 1	I-2010	Dead (2025)	Wild origin	Sexual reproduction	≈20 years	157.0	18,214.0
Female no. 2	I-2010	Dead (2026)	Wild origin	Sexual reproduction	≈21 years	151.5	20,142.0
Juvenile no. 1	B-2016	Dead	−	−	few days	−	−
Juvenile no. 2	B-2020	Dead	no. 1	Parthenogenetic	few days	33.7	131.5
Juvenile no. 3	B-2021	Dead (2024)	no. 2	Parthenogenetic	≈3 years	85.2	1248.0
Juvenile no. 4	B-2023	Dead	no. 1	Parthenogenetic	few days	32.5	129.7
Juvenile no. 5	B-2024	Dead	no. 2	Parthenogenetic	≤24 h	41.2	237.4
Juvenile no. 6	B-2024	Dead	no. 2	Parthenogenetic	−	26.9	133.1

TL: total length; W: weight.

**Table 2:** Microsatellite genotypes of *Mustelus mustelus* and parthenogenetic juveniles.

Locus	Allele Range (bp)	Female No. 1		Female No. 2		Juvenile No. 5		Juvenile No. 6		Function
McaB5	186–209	200	204	200	204	204	204	200	200	P+MA
Mh25	141–154	141	141	141	143	143	143	143	143	P+MA
McaB35	206–221	210	216	212	224	224	224	224	224	P+MA
MaD2X	179–185	185	185	183	185	183	183	185	185	P+MA
McaB26	224–229	224	224	229	229	n.d.	n.d.	229	229	MA
Gg20	280–282	280	280	280	280	280	280	280	280	NI
MaTJ5	157–159	159	159	159	159	159	159	159	159	NI
Gg4	198–199	198	198	198	198	198	198	198	198	NI
Mca33	194–200	200	200	200	200	200	200	200	200	NI
Mh1	201–203	203	203	203	203	203	203	203	203	NI
MaFYP	238–251	241	241	241	241	241	241	241	241	NI
Gg22	209–249	235	235	235	235	235	235	235	235	NI
Mh9	325–336	334	334	334	334	334	334	334	334	NI

n.d.: not detected (amplification failure after repeated attempts); P: parthenogenesis; MA: maternal assignment; NI: not informative.

## Data Availability

The original contributions presented in this study are included in the article/Supplementary Material. Further inquiries can be directed to the corresponding author.

## Supplementary Materials

The following supporting information can be downloaded at: https://www.mdpi.com/article/10.3390/ani16101423/s1, Table S1: Microsatellite loci, technical features, and primers are described in Marino et al. [22], and references therein.
