# Supplementary material for: Recurrent Alternate Parthenogenesis in the Common Smooth-Hound Shark (Mustelus mustelus) with Additional Cases and Further Evidence for a Putative Adaptive Reproductive Strategy
Source: Animals (Basel). 2026 May 7;16(10):1423. doi: 10.3390/ani16101423 (PMC13203237; doi:10.3390/ani16101423)
Supplement: Supplementary file 1 [file animals-16-01423-s001.zip › animals-4239324-supplementary.pdf]

Recurrent Alternate Parthenogenesis in the Common Smooth-Hound Shark (*Mustelus mustelus*) with Additional Cases and Further Evidence for a Putative Adaptive Reproductive Strategy

Simona Sciuto [1], Giuseppe Esposito [1,*], Flavio Gagliardi [2,3], Matteo Riccardo Di Nicola [1], Paolo Pastorino [1], Nadia Ruiu [2], Giulia Milanese [1], Nicole Kube [4], Oscar Di Santo [2,3], Marino Prearo [1], Pier Luigi Acutis [1] and Silvia Colussi [1]

[1] Istituto Zooprofilattico Sperimentale del Piemonte, Liguria e Valle d'Aosta, 10154 Turin, Italy; simona.sciuto@izsplv.it (S.S.); matteoriccardo.dinicola@izsplv.it (M.R.D.N.); paolo.pastorino@izsplv.it (P.P.); giulia.milanese@izsplv.it (G.M.); marino.prearo@izsplv.it (M.P.); pierluigi.acutis@izsplv.it (P.L.A.); silvia.colussi@izsplv.it (S.C.).
[2] Acquario di Cala Gonone, Cala Gonone, 08022 Dorgali, Italy
[3] Panaque s.r.l., 00144 Rome, Italy
[4] Ocean Museum Germany Foundation, Katharinenberg 14-20, 18439 Stralsund, Germany
* Correspondence: giuseppe.esposito@izsplv.it

SUPPLENTARY MATERIAL

Supplementary Table S1. Microsatellite loci, technical features, and primers are described in Marino et al. [22], and references therein.

Locus	Fluorophores	Size	Primer sequence (5′-3′)	Repeat size	PIC
McaB5	VIC	186 - 209	F: TAATCGACACGCAGTCATCG<br>R: AAGCTCCAATTCTCACTGTGC	2	0.375
Mh25	FAM	141 - 154	F: TGCAATAACCGTTCTGCGTC<br>R: TCACACCCGCAGTTAGATCC	2	0.359
McaB35	NED	206 - 221	F: AGTGCGTGCCAGTGTATGAG<br>R: GTTCTGCATGGGACGTGAC	2	0.524
MaD2X	FAM	179 - 185	F: ACCTGGCCCAAGAACTCTC<br>R: ACTGGTGATGTGTGGACCC	2	0.359
McaB26	FAM	224 - 229	F: ACTGTGGCACTGCATTCTGC<br>R: TGCATTTCAAAACCACTGGA	5	0.346
Gg20	PET	280 - 282	F: GACCAAGGGTCATCCAGAC<br>R: TCAGCTTGGGCAATTCCAG	2	0.000
MaTJ5	FAM	157 - 159	F: TGCCTCTGTTATGCCCCTC<br>R: GGGGTCGAGAAGCATGTTG	2	0.000
Gg4	PET	198 - 199	F: CTGGAATACATGCCGAGCAC<br>R: CCCGAAAGGTCTTAGTTCGC	2	0.000
Mca33	FAM	194 - 200	F: CATTTGAACCCCGACAGAAC<br>R: CCAAGTAAGGATGAGTGACACC	3	0.000
Mh1	VIC	201 - 203	F: GGAGGAGGGAAGCCTATGG<br>R: TCTCTGGCTCCATTCAGGG	2	0.000
MaFYP	FAM	238 - 251	F: TGGTTGCCGATACAGCAGG<br>R: CAAGCGCATGCACACTCAC	2	0.000
Gg22	VIC	209 - 249	F: TCCTGGGATGGCAACTTCG<br>R: AGGCCACCCAACTATCCTG	2	0.000
Mh9	NED	325 - 336	F: CAACCATCTTTACTACACTG<br>R: GATGGACCTCACATTTAACAC	2	0.000

PIC: Polymorphic Information Content.
